# A multicentre, randomised, open-label, parallel-group Phase 2b study of belotecan versus topotecan for recurrent ovarian cancer

**DOI:** 10.1038/s41416-020-01098-8

**Published:** 2020-09-30

**Authors:** Hee Seung Kim, Sang-Yoon Park, Chan-Yong Park, Young Tae Kim, Beob-Jong Kim, Yong Jung Song, Byoung-Gie Kim, Yong Beom Kim, Chi-Heum Cho, Jong-Hyeok Kim, Yong Sang Song

**Affiliations:** 1 Department of Obstetrics and Gynecology, Seoul National University College of Medicine, Seoul, 03080 Republic of Korea; 2 Center for Uterine Cancer, Research Institute and Hospital, National Cancer Center, Goyang, 10408 Republic of Korea; 3 Department of Obstetrics and Gynecology, Gil Medical Center, Gachon University of Medicine and Science, Incheon, 21565 Republic of Korea; 4 Department of Obstetrics and Gynecology, Institute of Women’s Medical Life Science, Yonsei University College of Medicine, Seoul, 03722 Republic of Korea; 5 Department of Obstetrics and Gynecology, Korea Cancer Center Hospital, Korea Institute of Radiological and Medical Sciences, Seoul, 01812 Republic of Korea; 6 Department of Obstetrics and Gynecology, Pusan National University School of Medicine, Yangsan, 50612 Republic of Korea; 7 Department of Obstetrics and Gynecology, Samsung Medical Center, Sungkyunkwan University School of Medicine, Seoul, 06351 Republic of Korea; 8 Department of Obstetrics and Gynecology, Seoul National University Bundang Hospital, Seongnam, 13620 Republic of Korea; 9 Department of Obstetrics and Gynecology, Dongsan Medical Center, Keimyung University, Daegu, 42601 Republic of Korea; 10 Department of Obstetrics and Gynecology, University of Ulsan College of Medicine, Asan Medical Center, Seoul, 05505 Republic of Korea

**Keywords:** Ovarian cancer, Chemotherapy

## Abstract

**Background:**

This Phase 2b study compared the efficacy and toxicity of belotecan and topotecan in recurrent ovarian cancer.

**Methods:**

Patients with platinum-sensitive recurrent or platinum-resistant recurrent ovarian cancer (PRROC) were randomised 1:1 to receive belotecan 0.5 mg/m^2^ or topotecan 1.5 mg/m^2^ for five consecutive days every 3 weeks. The primary endpoint was overall response rate (ORR); secondary endpoints were progression-free survival (PFS), overall survival (OS), and toxicity.

**Results:**

A total of 140 (belotecan, *n* = 71; topotecan, *n* = 69) and 130 patients (belotecan, *n* = 66; topotecan, *n* = 64) were included in the intention-to-treat (ITT) and per-protocol (PP) populations. ORR did not differ significantly between the belotecan and topotecan groups (ITT, 29.6% versus 26.1%; PP, 30.3% versus 25%). Although PFS did not differ between the groups, belotecan was associated with improved OS compared with topotecan in the PP population (39.7 versus 26.6 months; *P* = 0.034). In particular, belotecan showed longer OS in PRROC and non-high-grade serous carcinoma (non-HGSC; PP, adjusted hazard ratios, 0.499 and 0.187; 95% confidence intervals 0.255–0.977 and 0.039–0.895). Furthermore, there were no differences in toxicities between the two groups.

**Conclusions:**

Belotecan was not inferior to topotecan in terms of overall response for recurrent ovarian cancer.

**Clinical trial registration:**

NCT01630018.

## Introduction

Ovarian cancer has a poor prognosis, with a 5-year survival rate of <30% in stage IIIC–IV disease, with which more than two-thirds of patients are diagnosed.^1^ Despite a rate of complete response (CR) of 70–80% after primary treatment of advanced ovarian cancer, about 70% of patients ultimately relapse, and most of them die due to progression.^2^

Among patients showing disease recurrence, those with who relapse >6 months after the completion of primary chemotherapy are classified as platinum-sensitive recurrent ovarian cancer (PSROC), and platinum-based chemotherapy remains effective for them, with a response rate of up to 65%.^3^ In contrast, those with platinum-resistant recurrent ovarian cancer (PRROC), defined as recurrence ≤6 months after treatment, show a lower response rate of <30%, despite the use of non-platinum agents.^4^

Although targeted agents including bevacizumab and poly ADP ribose polymerase (PARP) inhibitors and immune-oncologic agents including programmed death receptor-1 (PD-1) and programmed death-ligand 1 (PD-L1) inhibitors have been introduced to overcome drug resistance, some of them are still combined with conventional cytotoxic drugs for treating recurrent ovarian cancer in clinical trials.

Belotecan is a semi-synthetic camptothecin analogue with the water-solubilising group at position 7 unlike topotecan, which inhibits the relegation of single-stranded DNA breaks by blocking topoisomerase I, and thereby disrupting DNA replication and inducing apoptosis of tumour cells.^5,6^ In preclinical studies, camptothecin including belotecan showed the inhibition of type 1 DNA topoisomerase in S phase cells, which caused the arrest of replication forks and led to cell killing.^7,8^ Moreover, these characteristics have been suggested to contribute to over twofold anti-tumour index of belotecan in comparison to topotecan,^5^ which can be related with better tumour response in ovarian cancer.^9^

In clinical studies, belotecan-based chemotherapy has also been reported to show tumour response rates of 53–78% in PSROC and 20–50% in PRROC,^10,11^ which are higher than the tumour responses seen with topotecan or topotecan plus cisplatin of 17–33% in PSROC,^12,13^ and 14–19% in PRROC.^14,15^ These findings suggest the possibility that belotecan may have greater potential than topotecan to improve prognosis in recurrent ovarian cancer. Thus, we performed a Phase 2b study to evaluate whether belotecan might be more effective than topotecan, with acceptable toxicity, in recurrent ovarian cancer, and thereby to assess the potential value of using it as a combination drug that could increase anti-cancer activity in the era of targeted and immuno-oncologic therapy.

## Materials and methods

### Study design

This was a multicentre, open-label, randomised, Phase 2b, non-inferiority study comparing the efficacy and toxicity of belotecan with that of topotecan in recurrent ovarian cancer (NCT01630018). The protocol was approved by Institutional Review Boards at Seoul National University Hospital, National Cancer Center, Gil Medical Center, Severance Hospital, Korea Cancer Center Hospital, Pusan National University Yangsan Hospital, Samsung Medical Center, Seoul National University Bundang Hospital, Dongsan Medical Center and Asan Medical Center before the start of the study. The manuscript was written in accordance with the CONSORT 2010 reporting guideline.

Eligibility criteria were as follows: ≥18 years of age; epithelial ovarian cancer; recurrent disease; measurable or non-measurable disease based on RECIST version 1.1^16^ or GCIG criteria;^17^ ECOG performance status ≤2; remaining life expectancy of >3 months; normal hepatic, renal and bone marrow function. We excluded patients with: active bacterial infection requiring intravenous antibiotics; brain metastasis; synchronous or metachronous malignancies other than epithelial ovarian cancer during the previous 5 years, except basal cell carcinoma of the skin or carcinoma in situ of the cervix that had been treated appropriately; and prior anti-cancer treatment within 4 weeks of enrolment.

### Treatment

All patients were assigned randomly 1:1 to receive either belotecan or topotecan. Belotecan 0.5 mg/m^2^ or topotecan 1.5 mg/m^2^ was administered as a 30-min infusion intravenously for five consecutive days every 3 weeks for six cycles or until disease progression, if earlier. Moreover, additional chemotherapy was administered according to the discretion of investigators if clinical benefit such as CR or partial response (PR) or stable disease was observed after the planed treatment.

When patients showed grade 3 or 4 haematologic or non-haematologic toxicities, the cycle of chemotherapy was delayed by up to 2 weeks. If grade 3 or 4 toxicities persisted after 2 weeks, that cycle of chemotherapy was cancelled, and the next cycle was administered according to the planned schedule. Dose reduction was considered when patients showed febrile neutropenia, grade 2 neutropenia, or grade 1 thrombocytopenia with delayed chemotherapy within 2 weeks. Dose reduction to belotecan 0.1 mg/m^2^/day or topotecan 0.25 mg/m^2^/day could be performed up to two times; administration was discontinued if an additional dose reduction was required.

### Endpoints

The primary endpoint was overall response rate (ORR), the ratio of patients with CR or PR among all patients. Secondary endpoints were progression-free survival (PFS; from the time of randomisation to the time of confirmation of disease progression or death) and overall survival (OS; from the time of randomisation to the time of confirmation of death or the end of the study). Rates of adverse events were also compared, based on CTCAE version 4.0.^18^

Efficacy was compared between belotecan and topotecan using the intention-to-treat (ITT) population (those who were originally allocated after randomisation and underwent one or more evaluation of response) and the per-protocol (PP) population (patients who completed the treated originally allocated). Safety was compared between belotecan and topotecan using the ITT population.

### Statistical analysis

For calculating the study sample size, ORR we selected as the primary endpoint because ORR was used as the primary endpoint in most previous trials with topotecan.^12–14,19,20^ This study was designed as a non-inferiority trial, and the ORR of topotecan reported for previous studies was considered when selecting the non-inferiority margin. The highest reported response rate for topotecan was 33%,^13^ and the lowest response rate was about 14% with 95% confidence interval (CI) of 8–19%.^19,20^ Thus, the non-inferiority margin was determined as 25%, calculated by subtracting the lowest margin of the CI of the lowest response rate (8%) from the highest response rate (33%). A total of 140 subjects, comprising 70 per group, were required, assuming the power of 80%, a type I error rate of 5% and a dropout rate of 20%.

Dichotomous data were analysed using the *χ*^2^ or Fisher’s exact test, and continuous variables were compared using the Student *t* or Mann–Whitney *U* test, comparing the experimental and control groups. Time-to-event variables were evaluated using Kaplan–Meier analysis with the log-rank or Breslow test. Independent prognostic factors were identified using Cox proportional hazard models with a hazard ratio (HR) and 95% CI. Statistical analyses were performed using SPSS software version 23.0 (Biostat Inc., Englewood, NJ, USA). *P* < 0.05 was considered significant.

## Results

### Population

**Fig. 1:**
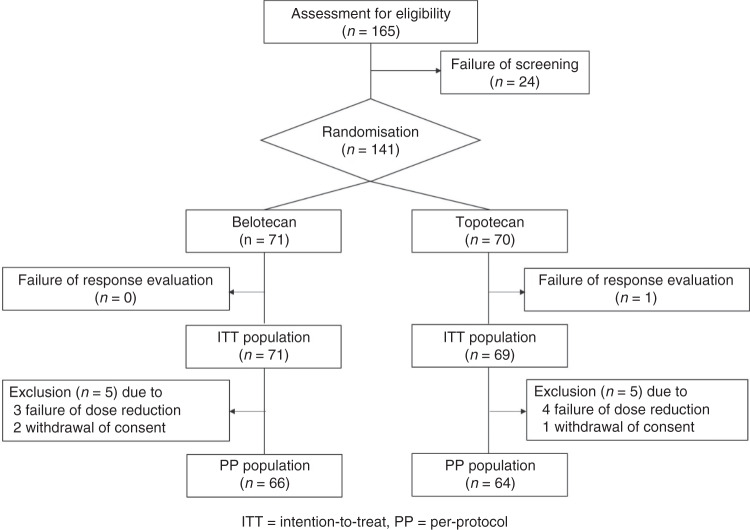
Patient disposition (CONSORT diagram).

A total of 141 patients were enrolled between January 2011 and June 2014, of whom 140 and 130 patients were included in ITT and PP populations after exclusion of 10 patients due to the protocol violation such as failure of dose reduction (*n* = 7) and withdrawal of consent (*n* = 3) (Fig. 1). Table 1 shows that clinicopathologic characteristics were similar between the belotecan and topotecan groups. Additional chemotherapy up to nine cycles was given in eight patients (5.7%) with high-grade serous carcinoma (HGSC). Among them, two with PSROC (2.8%) and four with PRROC (5.6%) received it in the belotecan group, whereas one with PSROC (1.4%) and one with PRROC (1.4%) received in the topotecan group. The rates of cancellation and dose reduction and delay of chemotherapy at each cycle were not different between the two groups (Supplementary Table 1).

**Table 1:** Clinicopathologic characteristics.

Characteristics	Belotecan (*n* = 71, %)	Topotecan (*n* = 69, %)	*P*-value
Age (mean, SD, years)	54.5 ± 10.6	56.9 ± 10.3	0.099
FIGO stage			0.294
I	6 (8.5)	4 (5.8)	
II	3 (4.2)	3 (4.3)	
III	36 (50.7)	47 (68.2)	
IV	26 (36.6)	15 (21.7)	
Histology			0.324
High-grade serous	49 (69.1)	57 (82.7)	
Endometrioid	6 (8.5)	2 (2.9)	
Mucinous	4 (5.6)	2 (2.9)	
Clear cell	4 (5.6)	5 (7.2)	
Undifferentiated	4 (5.6)	2 (2.9)	
Mixed	4 (5.6)^a^	1 (1.4)^b^	
Number of prior chemotherapy lines			0.480
1	37 (52.1)	34 (49.3)	
2	25 (35.2)	29 (42)	
3	7 (9.9)	6 (8.7)	
4	2 (2.8)	0 (0)	
Additional chemotherapy			0.275
No	65 (91.5)	67 (97.1)	
Yes	6 (8.5)	2 (2.9)	
Type of recurrence			0.408
PSROC	26 (36.6)	30 (43.5)	
PRROC	45 (63.4)	39 (56.5)	
Duration of follow-up (mean, SD, months)	18.7 ± 11.1	16.8 ± 9.7	0.297

*FIGO* International Federation of Gynecology and Obstetrics, *PRROC* platinum-resistant recurrent ovarian cancer, *PSROC* platinum-sensitive recurrent ovarian cancer, *SD* standard deviation.

^a^Three and one patient showed mixed high-grade serous/transitional cell carcinoma and mixed high-grade serous/clear cell/transitional cell carcinoma, respectively.

^b^One patient showed mixed high-grade serous/clear cell carcinoma.

### Efficacy

CR was not seen in the study, and ORR did not differ between the belotecan and topotecan groups in either the ITT (29.6% versus 26.1%, *P* = 0.645) or PP (30.3% versus 25.0%, *P* = 0.499) populations, which suggest that belotecan was not inferior to topotecan. In terms of the type of recurrence, ORR did not differ between the belotecan and topotecan groups among those with PSROC (ITT, 38.5% versus 33.3%; PP, 41.7% versus 34.6%; *P* > 0.05) or those with PRROC (ITT, 24.4% versus 20.5%; PP, 23.8% versus 18.4%; *P* > 0.05). Moreover, ORR also did not differ between the groups among those with high-grade serous carcinoma (HGSC; ITT, 28.6% versus 29.8%; PP, 28.3% versus 28.9%; *P* > 0.05) or non-HGSC (ITT, 31.8% versus 8.3%; PP, 33.3% versus 9.1%; *P* > 0.05; Table 2).

**Table 2:** Overall response rate.

Overall response rate	Belotecan	Topotecan	*P-*value
ITT population (*n*/*N*, %)			
All patients	21/71 (29.6)	18/69 (26.1)	0.645
Type of recurrence			
PSROC	10/26 (38.5)	10/30 (33.3)	0.783
PRROC	11/45 (24.4)	8/39 (20.5)	0.795
Histology			
HGSC	14/49 (28.6)	17/40 (29.8)	1.000
Non-HGSC	7/22 (31.8)	1/12 (8.3)	0.210
PP population (*n*/*N*, %)			
All patients	20/66 (30.3)	16/64 (25)	0.499
Type of recurrence			
PSROC	10/24 (41.7)	9/26 (34.6)	0.608
PRROC	10/42 (23.8)	7/38 (18.4)	0.556
Histology			
Serous	15/53 (28.3)	13.45 (28.9)	1.000
Non-serous	7/21 (33.3)	1/11 (9.1)	0.209

*HGSC* high-grade serous carcinoma, *ITT* intention-to-treat, *PP* per-protocol, *PRROC* platinum-resistant recurrent ovarian cancer, *PSROC* platinum-sensitive recurrent ovarian cancer.

**Fig. 2:**
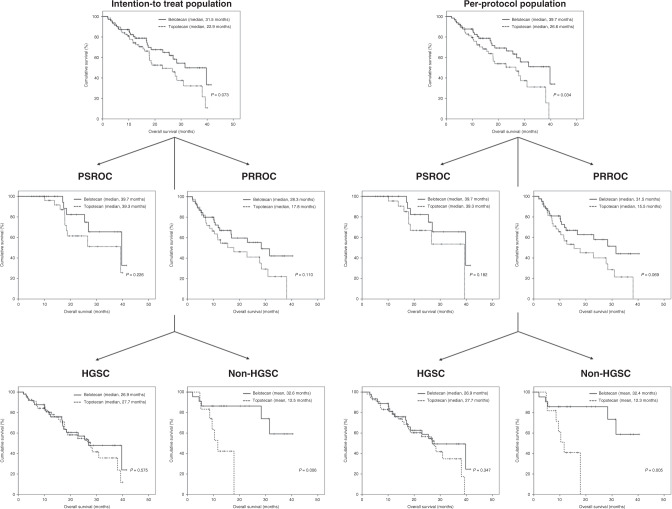
Comparison of overall survival between belotecan and topotecan in intention-to-treat and per-protocol populations. Subgroup analyses were conducted according to the type of recurrence (platinum-sensitive recurrent ovarian cancer, PSROC; platinum-resistant recurrent ovarian cancer, PRROC) and histology (high-grade serous carcinoma, HGSC; non-HGSC).

With respect to survival, there was no difference in PFS between the two groups (Supplementary Fig. 1). On the other hand, belotecan was associated with improved OS with marginal significance in the ITT population (median 31.5 versus 22.9 months, *P* = 0.073), with the relation becoming stronger in the PP population (median 39.7 versus 26.6 months; *P* = 0.034). There was no difference in OS between the two treatment groups among those with PSROC; however, belotecan was related to improved OS with marginal significance in the PP population among those with PRROC (median 31.5 versus 15.5 months, *P* = 0.069). Moreover, belotecan was also associated with prolonged OS in those with non-HGSC (ITT, mean 32.6 versus 12.5 months; PP, 32.4 versus 12.3 months; *P* < 0.05; Fig. 2).

In multivariate analyses, PSROC and additional chemotherapy were prognostic factors for PFS in patients with HGSC (ITT, adjusted HRs, 0.516 and 0.284; 95% CIs, 0.325–0.818 and 0.106–0.759; PP, 0.483 and 0.263; 0.297–0.785 and 0.097–0.711), and PSROC was the only prognostic factor for PFS in those with non-HGSC (ITT, adjusted HR, 0.285; 95% CI, 0.103–0.878; PP, 0.265; 0.086–0.813). However, belotecan was not a prognostic factor for PFS (Supplementary Tables 2 and 3).

On the other hand, PSROC was a prognostic factor for improving OS in those with HGSC (ITT, adjusted HR, 0.426; 95% CI, 0.221–0.820; PP, 0.421; 0.209–0.847) and those with non-HGSC (PP, adjusted HR, 0.156; 95% CI, 0.019–0.953). Moreover, belotecan was a favourable prognostic factor for improved OS in patients with PRROC (PP, adjusted HR 0.499; 95% CI 0.255–0.977; *P* = 0.043), and those with non-HGSC (ITT, adjusted HR, 0.185; 95% CI, 0.039–0.890; PP, 0.187; 0.039–0.895; Table 3).

**Table 3:** Multivariate analyses identifying prognostic factors for overall survival according to the type of recurrence, and histology.

	Type of recurrence					
	ITT population			PP population		
Factors	Adjusted HR	95% CI	*P*-value	Adjusted HR	95% CI	*P-*value
PSROC						
Age <55 years	0.868	0.295–2.554	0.797	1.372	0.418–4.505	0.602
HGSC	2.344	0.277–19.826	0.434	1.980	0.229–3.211	0.939
One prior chemotherapy	1.394	0.482–4.028	0.539	1.045	0.340–3.211	0.939
Additional chemotherapy	0.491	0.051–4.757	0.539	0.561	0.055–5.733	0.626
Belotecan	0.627	0.189–2.079	0.445	0.523	0.144–1.902	0.325
PRROC						
Age <55 years	1.226	0.640–2.347	0.539	1.243	0.639–2.417	0.521
HGSC	0.755	0.362–1.571	0.452	0.710	0.336–1.503	0.371
One prior chemotherapy	0.982	0.517–1.864	0.955	1.080	0.558– 2.091	0.818
Additional chemotherapy	1.294	0.376–4.458	0.683	1.413	0.405–4.937	0.588
Belotecan	0.558	0.294–1.061	0.075	0.499	0.255–0.977	0.043

	Histology					
	ITT population			PP population		
Factors	Adjusted HR	95% CI	*P*-value	Adjusted HR	95% CI	*P-*value
HGSC						
Age <55 years	1.097	0.588–2.049	0.771	1.278	0.663–2.463	0.464
PSROC	0.426	0.221–0.820	0.011	0.421	0.209–0.847	0.015
One prior chemotherapy	1.217	0.669–2.215	0.519	1.252	0.665–2.360	0.486
Additional chemotherapy	0.794	0.263–2.395	0.682	0.850	0.277–2.608	0.776
Belotecan	0.823	0.434–1.559	0.550	0.709	0.358–1.405	0.324
Non-HGSC						
Age <55 years	1.100	0.310–3.906	0.883	1.084	0.305–3.849	0.901
PSROC	0.136	0.017–1.079	0.059	0.156	0.019–0.953	0.040
One prior chemotherapy	0.744	0.206–2.913	0.705	0.755	0.200–2.850	0.678
Belotecan	0.185	0.039–0.890	0.035	0.187	0.039–0.895	0.036

*HGSC* high-grade serous carcinoma, *HR* hazard ratio, *CI* confidence interval, *ITT* intention-to-treat, *PP* per-protocol, *PRROC* platinum-resistant recurrent ovarian cancer, *PSROC* platinum-sensitive recurrent ovarian cancer.

In particular, 10 patients with endometrioid (*n* = 6) or clear cell carcinoma (*n* = 4) who received belotecan were alive, whereas 4 of 7 (57.1%) with endometrioid (*n* = 2) or clear cell carcinoma (*n* = 5) who received topotecan died during this study. When we performed subgroup analyses for the patients with endometrioid or clear cell carcinoma, there were no differences in clinicopathologic characteristics between the belotecan and topotecan groups (Supplementary Table 4). In terms of survival, belotecan was also associated with improved OS despite no difference in PFS between the two groups in either the ITT or PP populations (Supplementary Fig. 2).

### Safety

With respect to haematologic toxicity, grade 3 or 4 neutropenia and anaemia were observed commonly in the belotecan and topotecan groups (grade 3: 64.8% versus 53.6%, and 15.5% versus 20.3%; grade 4: 52.1% vs. 39.1%, and 1.4% versus 0%). In terms of non-haematologic toxicity, grade 3 diarrhoea and ileus were relatively common in the belotecan and topotecan groups (4.2% versus 0.0%, and 0.0% versus 2.9%). However, there were no differences in both haematologic and non-haematologic toxicities between the belotecan and topotecan groups (Table 4).

**Table 4:** Adverse events.

Adverse event	Grade 1			Grade 2			Grade 3			Grade 4		
	Belotecan (*n* = 71, %)	Topotecan (*n* = 69, %)	*P-* value	Belotecan (*n* = 71, %)	Topotecan (*n* = 69, %)	*P-* value	Belotecan (*n* = 71, %)	Topotecan (*n* = 69, %)	*P-* value	Belotecan (*n* = 71, %)	Topotecan (*n* = 69, %)	*P-* value
Neutrophil count decreased	1 (1.4)	0 (0)	1.000	34 (47.9)	38 (55.1)	0.395	46 (64.8)	37 (53.6)	0.179	37 (52.1)	27 (39.1)	0.123
Anaemia	3 (4.2)	3 (4.3)	1.000	19 (26.8)	22 (31.9)	0.505	11 (15.5)	14 (20.3)	0.459	1 (1.4)	0 (0)	1.000
Platelet count decreased	3 (4.2)	5 (7.2)	0.490	5 (7)	8 (11.6)	0.354	6 (8.5)	5 (7.2)	0.791	3 (4.2)	2 (2.9)	1.000
Febrile neutropenia	–	–	–	–	–	–	1 (1.4)	1 (1.4)	1.000	0 (0)	0 (0)	–
Nausea	18 (25.4)	25 (36.2)	0.163	40 (54.1)	34 (49.3)	0.568	1 (1.4)	1 (1.4)	1.000	0 (0)	0 (0)	–
Vomiting	10 (14.1)	9 (13)	1.000	8 (11.3)	14 (20.3)	0.143	1 (1.4)	0 (0)	1.000	0 (0)	0 (0)	–
Anorexia	14 (19.7)	12 (17.4)	0.723	6 (8.5)	10 (14.5)	0.261	0 (0)	1 (1.4)	0.493	0 (0)	0 (0)	–
Aspartate aminotransferase increased	0 (0)	1 (1.4)	0.493	0 (0)	1 (1.4)	0.493	0 (0)	1 (1.4)	0.493	0 (0)	0 (0)	–
Diarrhoea	4 (5.6)	4 (5.8)	0.967	4 (5.6)	2 (2.9)	0.681	3 (4.2)	0 (0)	0.245	0 (0)	0 (0)	–
Ileus	0 (0)	0 (0)	–	1 (1.4)	1 (1.4)	1.000	0 (0)	2 (2.9)	0.241	0 (0)	0 (0)	–
Hypocalcemia	0 (0)	0 (0)	–	1 (1.4)	0 (0)	1.000	1 (1.4)	0 (0)	1.000	0 (0)	0 (0)	–
Hypokalemia	1 (1.4)	0 (0)	1.000	0 (0)	1 (1.4)	0.493	0 (0)	1 (1.4)	0.493	0 (0)	0 (0)	–
Fatigue	5 (7)	6 (8.7)	0.716	5 (7)	6 (8.7)	0.716	0 (0)	1 (1.4)	0.493	0 (0)	0 (0)	–
Syncope	0 (0)	0 (0)	–	0 (0)	0 (0)	–	1 (1.4)	0 (0)	1.000	0 (0)	0 (0)	–
Back pain	1 (1.4)	0 (0)	1.000	1 (1.4)	0 (0)	1.000	1 (1.4)	0 (0)	1.000	0 (0)	0 (0)	–
Lung infection	0 (0)	0 (0)	–	1 (1.4)	0 (0)	1.000	1 (1.4)	0 (0)	1.000	0 (0)	0 (0)	–
Rash acneiform	0 (0)	3 (4.3)	0.117	0 (0)	1 (1.4)	0.493	0 (0)	1 (1.4)	0.493	0 (0)	0 (0)	–
Hypotension	0 (0)	0 (0)	–	0 (0)	0 (0)	–	0 (0)	1 (1.4)	0.493	0 (0)	0 (0)	–

## Discussion

Topoisomerase I inhibitors are popular anti-cancer drugs that interrupt the ligation step of the cancer cell cycle, generating single or double-stranded breaks that harm the integrity of the genome, leading to apoptosis or cell death. Although bevacizumab or PARP inhibitors combined with taxane- and platinum-based chemotherapy have been shown to be effective for treating PSROC,^21,22^ the cytotoxic effects of topoisomerase I inhibitors are still important for combination therapy for treating PRROC in the era of targeted and immuno-oncologic therapy.^23–26^

In this study, belotecan showed similar ORR, PFS, and toxicity to topotecan. In particular, belotecan showed similar efficacy to topotecan as a topoisomerase I inhibitor for improving OS in patients with PSROC and HGSC, as indicated by there being no difference in OS between the two treatments in these subgroups. However, the ORR of belotecan was relatively low when compared with the result of our previous report where it was combined with cisplatin (38.5–41.7% versus 78%), suggesting that belotecan combined with cisplatin may be more effective than belotecan alone for PSROC.^11^

On the other hand, belotecan increased OS by 10.5–16 months compared with topotecan in patients with PRROC. These findings are meaningful when we considered that patients who received >2 prior chemotherapeutic regimens accounted for 10% of all patients, unlike the AURELIA trial, from which they were excluded,^27^ and that targeted or immune-oncologic drugs had not been administered in all patients after disease progression because these agents have been permitted since May 2005 in our country.^28^ Although there is no clear evidence indicating why belotecan may be associated with improved OS in PRROC, a preclinical study, where anti-tumour activity was compared between belotecan and topotecan without the addition of bevacizumab, has reported that anti-tumour activity of topotecan was not observed after 240 min, whereas it was seen with belotecan for more than 250 min in various types of cancer cell lines,^5^ suggesting that the relatively long-lasting anti-tumour activity of belotecan can contribute to the improved OS when compared to topotecan.

Another interesting point is that belotecan was also associated with improved OS in patients with non-HGSC in the current study. Although we cannot explain this finding on the basis of evidence, we can suggest the hypothesis that belotecan may have more potential to increase T-cell-mediated cytotoxicity than topotecan because preclinical studies reported that topoisomerase I inhibitors may increase T-cell-mediated cytotoxicity depending on the upregulation of tumour protein 53-inducible nuclear protein 1 (TP53INP1) to positively regulate tumour cell apoptosis in melanoma cells.^29,30^ Especially, we found that patients with endometrioid or clear cell carcinoma who received belotecan were alive despite the death in 57.1% of the patients who received topotecan, suggesting improved OS by belotecan. Considering that TP53INP1 is upregulated in most of the patients with non-HGSC, the result supports the hypothesis that belotecan can further enhance T-cell-mediated cytotoxicity in comparison to topotecan, which should be proven in basic research.

This study has several limitations that mean further trials are required to validate the findings. First, the result that belotecan was not inferior to topotecan in terms of overall response should be further investigated in well-designed studies because this study included both patients with PSROC and those with PRROC. Since patients with PSROC may show better ORR than those with PRROC in general, ORR between belotecan and topotecan should be compared in trials including each group. Second, we set ORR as the primary endpoint instead of OS and designed the study as a Phase 2b trial. More clinical trials focused on OS is needed to prove that belotecan can improve OS. Third, patients with recurrent ovarian cancer were recruited heterogeneously regardless of the type of recurrence and histology. If belotecan may be more effective than topotecan, specifically in patients with PRROC or non-HGSC, new trials where only these patients are enrolled will be needed to prove this. Fourth, the potential of belotecan on better T-cell immunity or tumour response should be proven in basic research.

Despite these limitations, the study is meaningful because it suggests that belotecan maybe not inferior to topotecan in terms of overall response for recurrent ovarian cancer. Therefore, these results allow us to use belotecan for treating recurrent ovarian cancer in anticipation of treatment response comparable to topotecan.

## Supplementary information

Supplementary table 1

Supplementary table 2

Supplementary table 3

Supplementary table 4

Supplementary figure 1

Supplementary figure 2

## Data Availability

A central manager had independent access to all data and materials. The central manager managed the database by sending queries about errors entered from each institution and having principal investigators or clinical research nurses make corrections to the entered data.
