# Supplementary material for: A multicentre, randomised, open-label, parallel-group Phase 2b study of belotecan versus topotecan for recurrent ovarian cancer
Source: Br J Cancer. 2020 Sep 30;124(2):375–82. doi: 10.1038/s41416-020-01098-8 (PMC7853132; doi:10.1038/s41416-020-01098-8)
Supplement: Supplementary file 1 — Supplementary table 1 [file 41416_2020_1098_MOESM1_ESM.docx]

Supplementary table 1. Comparison of cancellation, dose reduction and delay of cycles between belotecan and topotecan

| Parameter | Belotecan | Topotecan | *P* value |
| --- | --- | --- | --- |
| Cancellation (n/N, %) |  |  |  |
| Cycle 1 (n=140) | 4/71 (5.6) | 4/69 (5.8) | 1.000 |
| Cycle 2 (n=130) | 2/66 (3) | 4/64 (6.2) | 0.437 |
| Cycle 3 (n=92) | 1/47 (2.1) | 4/45 (8.9) | 0.198 |
| Cycle 4 (n=82) | 2/42 (4.5) | 4/40 (10) | 0.418 |
| Cycle 5 (n=68) | 3/35 (8.6) | 4/33 (12.1) | 0.705 |
| Cycle 6 (n=68) | 3/35 (8.6) | 0/33 (0) | 0.239 |
| Dose reduction (n, %) |  |  |  |
| Cycle 1 (n=140) | 0/71 (0) | 0/69 (0) | – |
| Cycle 2 (n=130) | 3/66 (4.5) | 9/64 (14.1) | 0.074 |
| Cycle 3 (n=92) | 3/47 (4.2) | 0/45 (0) | 0.242 |
| Cycle 4 (n=82) | 0/42 (0) | 0/40 (0) | – |
| Cycle 5 (n=68) | 1/35 (1.4) | 0/33 (0) | 1.000 |
| Cycle 6 (n=68) | 0/35 (0) | 0/33 (0) | – |
| Delay (mean, SD, days) |  |  |  |
| Cycle 1 (n=140) | 7 ± 3.6 | 8.3 ± 4.2 | 0.092 |
| Cycle 2 (n=130) | 7.3 ± 3.6 | 7.2 ± 3.8 | 0.916 |
| Cycle 3 (n=92) | 7 ± 3.6 | 8.5 ± 3.1 | 0.123 |
| Cycle 4 (n=82) | 7.1 ± 3.4 | 8.3 ± 4.3 | 0.299 |
| Cycle 5 (n=68) | 6.6 ± 3.3 | 6.7 ± 1.5 | 0.971 |
| Cycle 6 (n=68) | 7.2 ± 2.6 | 7.8 ± 3 | 0.623 |

Abbreviation: SD, standard deviation.

Cancellation: the cycle of chemotherapy was delayed by up to 2 weeks when patients showed grade 3 or 4 hematologic or non-hematologic toxicities.

Dose reduction: dose reduction was considered when patients showed febrile neutropenia, grade 2 neutropenia, or grade 1 thrombocytopenia with delayed chemotherapy within 2 weeks. Dose reduction to belotecan 0.1 mg/ m^2^/day or topotecan 0.25 mg/ m^2^/day could be performed up to two times, and administration was discontinued if an additional dose reduction was required.

Delay: the cycle of chemotherapy was cancelled if grade 3 or 4 toxicities persisted after 2 weeks, and the next cycle was administered according to the planned schedule.
