# Supplementary material for: A multicentre, randomised, open-label, parallel-group Phase 2b study of belotecan versus topotecan for recurrent ovarian cancer
Source: Br J Cancer. 2020 Sep 30;124(2):375–82. doi: 10.1038/s41416-020-01098-8 (PMC7853132; doi:10.1038/s41416-020-01098-8)
Supplement: Supplementary file 2 — Supplementary table 2 [file 41416_2020_1098_MOESM2_ESM.docx]

Supplementary table 2. Multivariate analyses identifying prognostic factors for progression-free survival stratified by type of recurrence

| PSROC | ITT population | | | PP population | | |
| --- | --- | --- | --- | --- | --- | --- |
| Factor | Adjusted HR | 95% CI | *P* value | Adjusted HR | 95% CI | *P* value |
| Age <55 years | 0.991 | 0.483 – 2.032 | 0.980 | 0.966 | 0.453 – 2.060 | 0.928 |
| HGSC | 1.313 | 0.486 – 3.547 | 0.591 | 1.519 | 0.510 – 4.528 | 0.453 |
| One prior chemotherapy | 1.159 | 0.587 – 2.289 | 0.671 | 1.366 | 0.657 – 2.839 | 0.404 |
| Additional chemotherapy | 0.257 | 0.050 – 1.316 | 0.103 | 0.234 | 0.044 – 1.240 | 0.088 |
| Belotecan | 0.983 | 0.454 – 2.128 | 0.966 | 0.882 | 0.393 – 1.976 | 0.760 |
| PRROC | ITT population | | | PP population | | |
| Factor | Adjusted HR | 95% CI | *P* value | Adjusted HR | 95% CI | *P* value |
| Age <55 years | 1.443 | 0.882 – 2.363 | 0.144 | 1.378 | 0.829 – 2.292 | 0.216 |
| HGSC | 0.962 | 0.546 – 1.694 | 0.892 | 0.999 | 0.560 – 1.783 | 0.997 |
| One prior chemotherapy | 1.071 | 0.665 – 1.724 | 0.778 | 1.049 | 0.642 – 1.714 | 0.849 |
| Additional chemotherapy | 0.323 | 0.097 – 1.079 | 0.066 | 0.315 | 0.094 – 1.058 | 0.062 |
| Belotecan | 0.977 | 0.592 – 1.613 | 0.929 | 0.941 | 0.561 – 1.578 | 0.818 |

Abbreviations: HGSC, high-grade serous carcinoma; HR, hazard ratio; CI, confidence interval; ITT, intention-to-treat; PP, per-protocol; PRROC, platinum-resistant recurrent ovarian cancer; PSROC, platinum-sensitive recurrent ovarian cancer.
