# Supplementary material for: A multicentre, randomised, open-label, parallel-group Phase 2b study of belotecan versus topotecan for recurrent ovarian cancer
Source: Br J Cancer. 2020 Sep 30;124(2):375–82. doi: 10.1038/s41416-020-01098-8 (PMC7853132; doi:10.1038/s41416-020-01098-8)
Supplement: Supplementary file 3 — Supplementary table 3 [file 41416_2020_1098_MOESM3_ESM.docx]

Supplementary table 3. Multivariate analyses identifying prognostic factors for progression-free survival according to histology

| HGSC | ITT population | | | PP population | | |
| --- | --- | --- | --- | --- | --- | --- |
| Factor | Adjusted HR | 95% CI | *P* value | Adjusted HR | 95% CI | *P* value |
| Age <55 years | 0.969 | 0.610 – 1.540 | 0.895 | 0.903 | 0.554 – 1.472 | 0.683 |
| PSROC | 0.516 | 0.325 – 0.818 | 0.005 | 0.483 | 0.297 – 0.785 | 0.003 |
| One prior chemotherapy | 0.980 | 0.631 – 1.522 | 0.927 | 0.971 | 0.613 – 1.539 | 0.900 |
| Additional chemotherapy | 0.284 | 0.106 – 0.759 | 0.012 | 0.263 | 0.097 – 0.711 | 0.008 |
| Belotecan | 0.786 | 0.486 – 1.217 | 0.326 | 0.689 | 0.416 – 1.141 | 0.148 |
| Non-HGSC | ITT population | | | PP population | | |
| Factor | Adjusted HR | 95% CI | *P* value | Adjusted HR | 95% CI | *P* value |
| Age <55 years | 1.648 | 0.818 – 6.887 | 0.106 | 2.388 | 0.904 – 6.313 | 0.079 |
| PSROC | 0.285 | 0.103 – 0.787 | 0.015 | 0.265 | 0.086 – 0.813 | 0.020 |
| One prior chemotherapy | 1.723 | 0.692 – 4.294 | 0.243 | 1.795 | 0.699 – 4.610 | 0.224 |
| Belotecan | 1.564 | 0.605 – 4.044 | 0.356 | 0.623 | 0.236 – 1.644 | 0.339 |

Abbreviations: HGSC, high-grade serous carcinoma; HR, hazard ratio; CI, confidence interval; ITT, intention-to-treat; PP, per-protocol; PRROC, platinum-resistant recurrent ovarian cancer; PSROC, platinum-sensitive recurrent ovarian cancer.
