# Supplementary material for: A multicentre, randomised, open-label, parallel-group Phase 2b study of belotecan versus topotecan for recurrent ovarian cancer
Source: Br J Cancer. 2020 Sep 30;124(2):375–82. doi: 10.1038/s41416-020-01098-8 (PMC7853132; doi:10.1038/s41416-020-01098-8)
Supplement: Supplementary file 4 — Supplementary table 4 [file 41416_2020_1098_MOESM4_ESM.docx]

Supplementary table 4. Clinicopathologic characteristics in patients with endometrioid or clear cell carcinoma

| Characteristics | Belotecan (n=10, %) | Topotecan (n=7, %) | *P* value |
| --- | --- | --- | --- |
| Age (median, range, years) | 52 (43, 61) | 52 (38, 60) | 0.624 |
| FIGO stage |  |  | 1.000 |
| I | 3 (30) | 2 (28.6) |  |
| II | 1 (10) | 0 (0) |  |
| III | 5 (50) | 2 (28.6) |  |
| IV | 1 (10) | 3 (42.9) |  |
| Histology |  |  | 0.335 |
| Endometrioid | 6 (60) | 2 (28,6) |  |
| Clear cell | 4 (40) | 5 (71.4) |  |
| Number of prior chemotherapy lines |  |  | 0.606 |
| 1 | 5 (50) | 3 (42.9) |  |
| 2 | 4 (40) | 4 (57.1) |  |
| 3 | 1 (10) | 0 (0) |  |
| Type of recurrence |  |  | 0.637 |
| PSROC | 6 (60) | 3 (42.9) |  |
| PRROC | 4 (40) | 4 (57.1) |  |

Abbreviations: FIGO, International Federation of Gynecology and Obstetrics; PRROC, platinum-resistant recurrent ovarian cancer; PSROC, platinum-sensitive recurrent ovarian cancer.
