# Supplementary figures and images for: A multicentre, randomised, open-label, parallel-group Phase 2b study of belotecan versus topotecan for recurrent ovarian cancer
Source: Br J Cancer. 2020 Sep 30;124(2):375–82. doi: 10.1038/s41416-020-01098-8 (PMC7853132; doi:10.1038/s41416-020-01098-8)

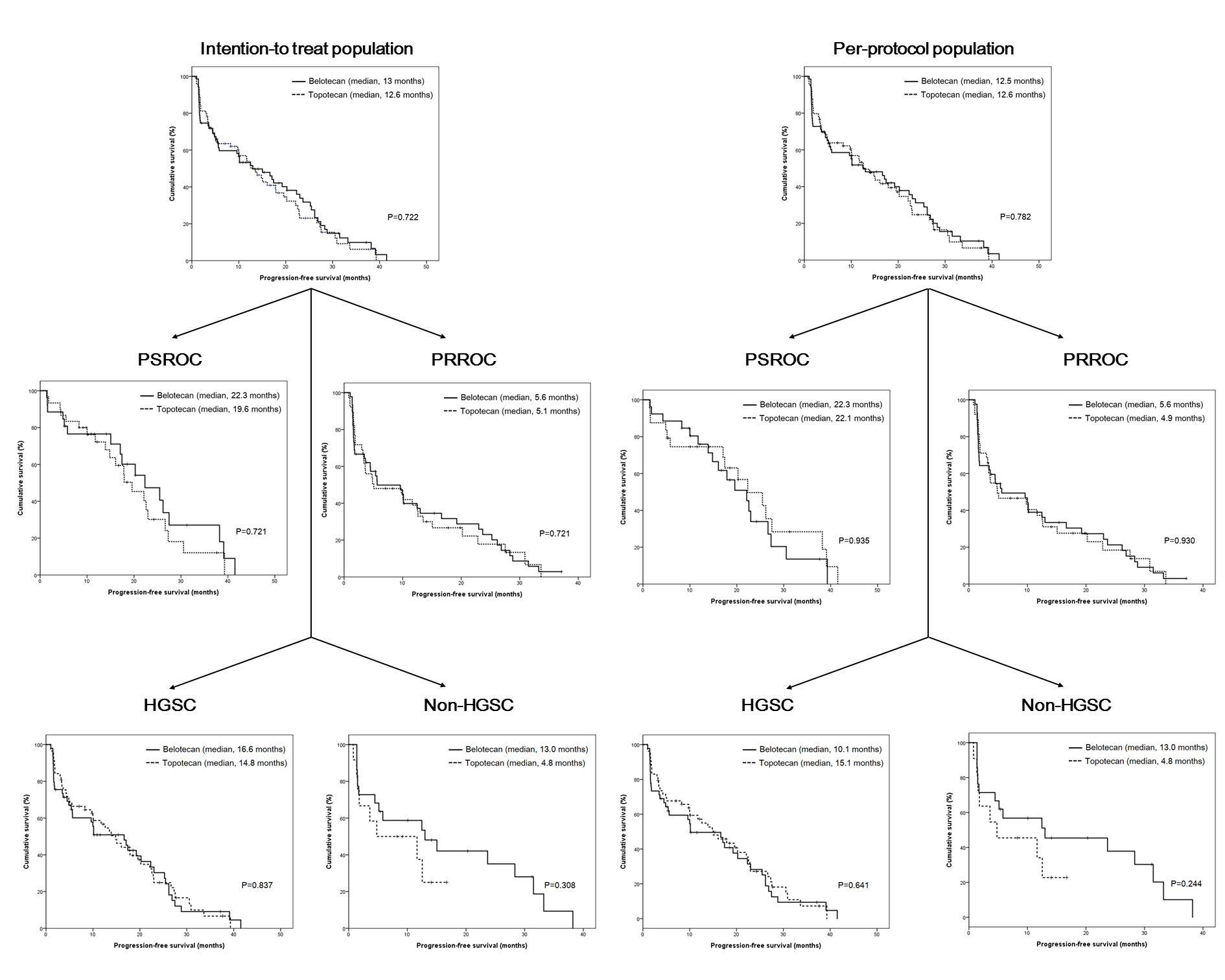

Supplement: Supplementary file 5 — Supplementary figure 1 [file 41416_2020_1098_MOESM5_ESM.tif]

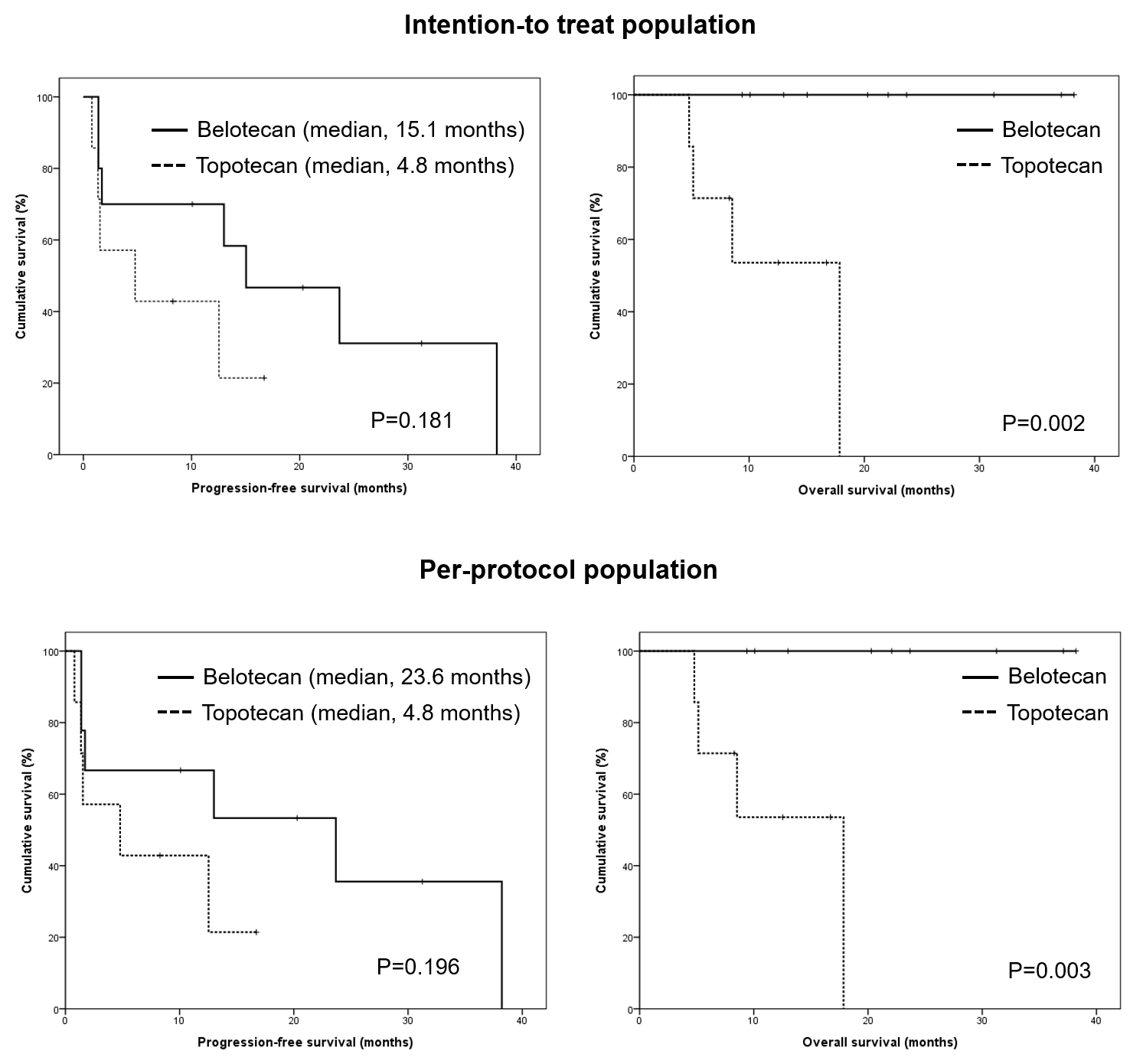

Supplement: Supplementary file 6 — Supplementary figure 2 [file 41416_2020_1098_MOESM6_ESM.tif]
